# Methamphetamine regulation of activity and topology of ventral midbrain networks

**DOI:** 10.1371/journal.pone.0222957

**Published:** 2019-09-19

**Authors:** Douglas R. Miller, Joseph J. Lebowitz, Dylan T. Guenther, Alexander J. Refowich, Carissa Hansen, Andrew P. Maurer, Habibeh Khoshbouei

**Affiliations:** 1 Department of Neuroscience, University of Florida, Gainesville, FL, United States of America; 2 McKnight Brain Institute, University of Florida, Gainesville, FL, United States of America; 3 Department of Biomedical Engineering, University of Florida, Gainesville, FL, United States of America; 4 Department of Civil and Coastal Engineering, University of Florida, Gainesville, FL, United States of America; University of Kentucky, UNITED STATES

## Abstract

The ventral midbrain supports a variety of functions through the heterogeneity of neurons. Dopaminergic and GABA neurons within this region are particularly susceptible targets of amphetamine-class psychostimulants such as methamphetamine. While this has been evidenced through single-neuron methods, it remains unclear whether and to what extent the local neuronal network is affected and if so, by which mechanisms. Both GABAergic and dopaminergic neurons were heavily featured within the primary ventral midbrain network model system. Using spontaneous calcium activity, our data suggest methamphetamine decreased total network output via a D_2_ receptor-dependent manner. Over culture duration, functional connectivity between neurons decreased significantly but was unaffected by methamphetamine. However, across culture duration, exposure to methamphetamine significantly altered changes in network assortativity. Here we have established primary ventral midbrain networks culture as a viable model system that reveals specific changes in network activity, connectivity, and topology modulation by methamphetamine. This network culture system enables control over the type and number of neurons that comprise a network and facilitates detection of emergent properties that arise from the specific organization. Thus, the multidimensional properties of methamphetamine can be unraveled, leading to a better understanding of its impact on the local network structure and function.

## Introduction

The ventral midbrain neurons are one of the main targets of amphetamines. Amphetamine-class drugs have been shown to increase extracellular dopamine levels by stimulation of firing activity of dopamine neurons and via stimulation of dopamine efflux through the dopamine transporter [1]. Once in the extracellular space, dopamine rapidly diffuses from the site of release and acts upon both pre- and postsynaptic receptors [2–4]. Activation of presynaptic D_2_ receptors in turn decreases firing activity of dopaminergic neurons and activation of postsynaptic D_2_ receptors decreases postsynaptic neuronal activity [5]. Importantly, the majority of ventral midbrain neurons are non-dopaminergic neurons [6], but many of which express D_2_ receptors [7] that are modulated secondary to methamphetamine stimulation of dopamine release. While these effects have been extensively investigated in individual neurons, it remains unexplored how these activities affect the functional connectivity and topological network features.

Network analysis provides the necessary tools to determine how patterns emerge from paired neuronal activity, how they segregate and integrate information, and how specific regional composition and conditions predispose the network to failure [8–11]. For instance, in a previous study, neurons were plated in the absence of adhesion proteins, forcing neurons to grow in dense clusters. The activity of the dense clusters persisted in the presence of an excitatory neurotransmission inhibitor, or when neurons were exposed to light radiation. The observed persistence in dense clusters was contrasted to observations of cultures where the spatial distribution of neurons was homogenous, and activity decayed quickly under both conditions. In the clustered cultures, the networks were positively assortative while the homogenous cultures were approximately neutral, similar to other studies of cultured cortical networks [12,13]. Network analysis has also revealed unique signatures in hippocampal slices. In both acute and cultured hippocampal slice networks, gamma-aminobutyric acid (GABA) neurons function as network hubs, which orchestrate network synchrony [14,15]. The advantages of network science approaches can therefore be leveraged to investigate whether ventral midbrain network topology changes over time in culture or restructures during methamphetamine exposure.

GABA neurons are the major inhibitory interneurons in the ventral midbrain [16]. These neurons both project within and outside of the ventral midbrain. Unlike dopaminergic neurons, the basal physiological properties of GABAergic neurons are less understood [7]. Nevertheless, they are implicated in methamphetamine-mediated responses [17,18]. Previous work showed that following a single injection of methamphetamine, ventral midbrain GABA neurons exhibited depression of GABA_B_ receptor signaling [18]. Furthermore, previous studies in organotypic hippocampal culture and acute cortical slices showed that exposure to neuromodulators such as acetylcholine or domoic acid inhibition of GABA neurons was sufficient to disrupt and reorganize network connectivity [19,20]. However, it is unclear whether the activity of GABA neurons, their functional connectivity, and/or network topology is altered during methamphetamine exposure. This study is aimed to uncover the mechanisms by which the functional connectivity and structure of cultured GABAergic networks is modulated by methamphetamine. Our goal is to establish a standard methodology to investigate neuronal network topology and to use this model to determine how methamphetamine regulates ventral midbrain network activity.

In this study, we have exploited spontaneous calcium activity, a proxy of neuronal activity [21], to determine the functional connectivity and network topology of ventral midbrain network culture. We determined how methamphetamine dysregulates network activity and impacts topological measurements, which can provide clues on how methamphetamine modulation of neuronal activity regulates functional connectivity relationships and topology of ventral midbrain neuronal networks. Our work represents the first investigation, to our knowledge, into the basal network topology of the ventral midbrain with single neuron resolution and further demonstrates the multitude of effects by methamphetamine from individual neurons to the constellation of the local network.

## Results and discussion

### Overview of network analysis and metrics in this study

**Fig 1:**
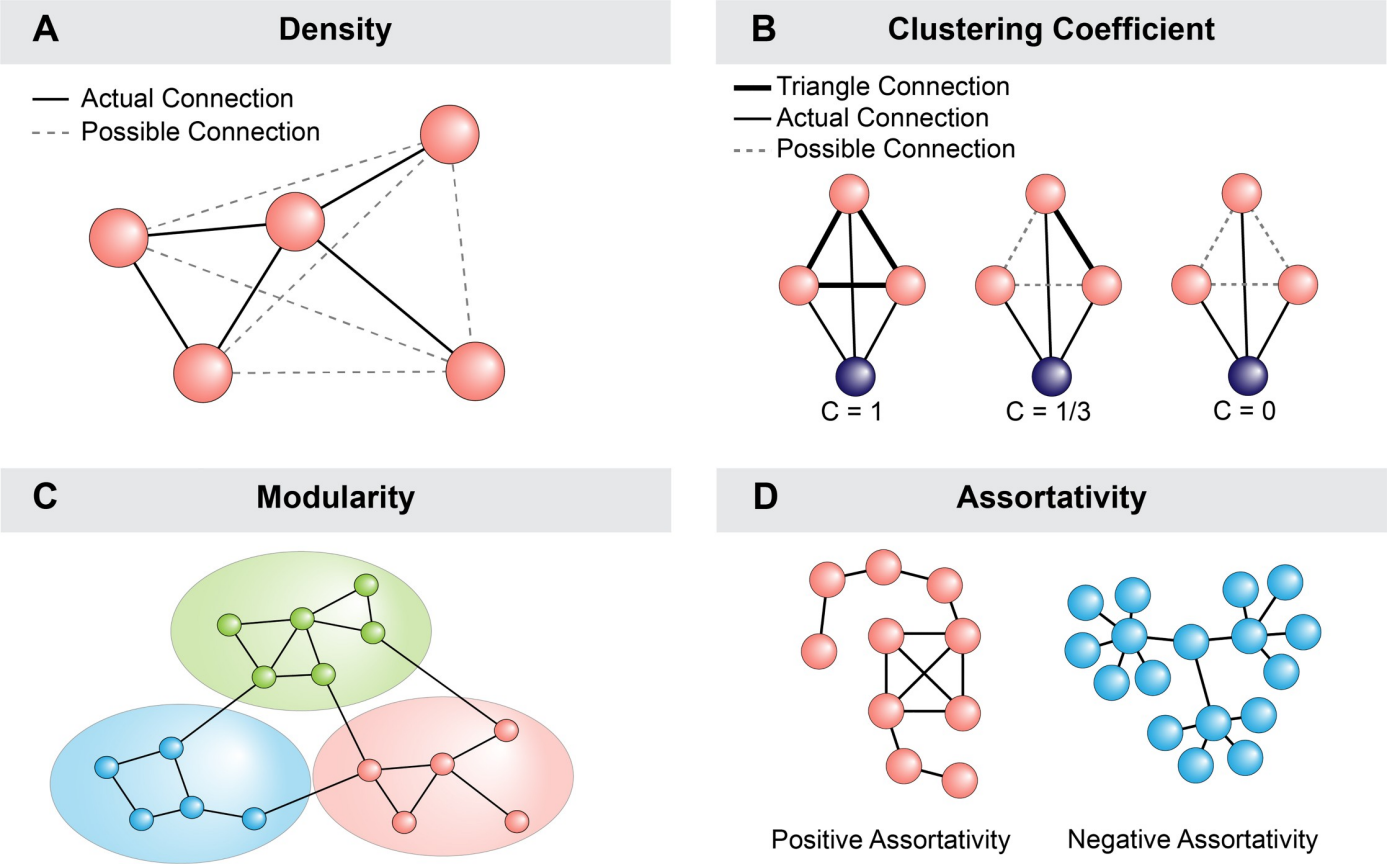
Overview of network metrics. This study utilizes network calculations of density, clustering coefficient, modularity and assortativity. (A) Density represents the actual connections to the total possible connections. Real-world networks are typically sparsely connected, conferring a low density. (B) Clustering coefficient determines the interconnectedness around each neuron by measuring the fraction of complete triangle connections. This metric extends from pair-wise connections to connectivity patterns that have implications on information propagation. (C) Modularity extends upon clustering by segregating the network into subgroups of high interconnectedness. Increases or decreases in modularity index are indicative of changes in community structure. (D) Assortativity evaluates preferential attachment between neurons of similar connectivity (positive) or dissimilar (negative).

Methamphetamine is a highly addictive drug that increases intracellular calcium concentration to modulate neuronal activity [22]. How methamphetamine modulates the communication across neurons in a network is unclear. To determine how methamphetamine-modulation of single neurons affects functional connectivity relationships and topology of ventral midbrain neuronal networks, we employed network analysis. Functional connectivity between two neurons was defined as correlated activity [23] measured by the Spearman’s rank correlation [12]. Functional connectivity was then used to calculate network density, clustering coefficient, modularity, and assortativity as representative metrics before and during methamphetamine exposure. Density is defined as the fraction of actual connections relative to total possible connections [9]. Clustering coefficient extends upon pair-wise correlations by determining the fraction of complete triangular connections around a neuron. Clustering coefficient is commonly applied to represent patterns of connectivity not evident from pair-wise correlations [24–26]. To identify the potential existence of individual subnetworks within the larger neuronal network, the modularity index (Q) was calculated. Finally, it has been shown that network topology may confer susceptibility to loss of critical nodes [9,12]. Therefore, we calculated assortativity as a general estimate of network resiliency. In general, a positive assortativity refers to preferential connectivity of neurons with similar connectivity [10]. For example, neurons with 10 connections are more likely to be connected to other neurons with multiple connections rather than those with a few connections. Negative assortativity is conventionally characterized by preferential connectivity to neurons with dissimilar connectivity [10]. For instance, neurons with 10 connections are more likely to be connected to neurons with 1 to 2 connections. Therefore, in certain situations, positive assortativity confers resiliency of the network, which enables compensation following neuronal loss (Fig 1).

### Construction and characterization of ventral midbrain neuronal networks

**Fig 2:**
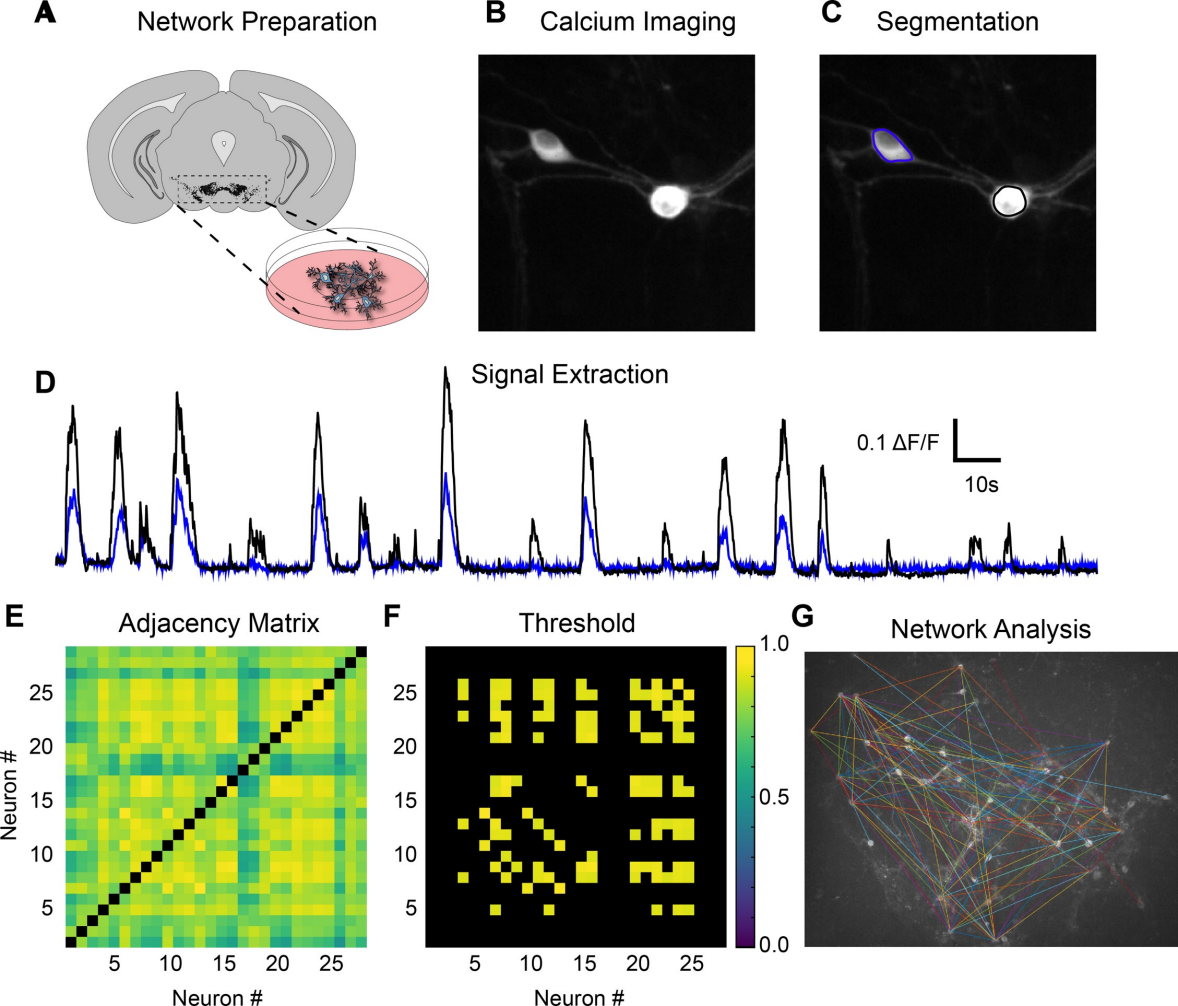
Analysis of functional connectivity in neuronal networks using calcium activity. (A) Midbrain networks cultures are prepared from P0-P1 mice by extraction of the ventral midbrain (dotted box) and viral production of GCaMP6f. (B) Spontaneous calcium activity is monitored in a 10X imaging field at 10 Hz. (C) Neuronal somas are segmented based on visual activity. (D) Time-resolved calcium signals are extracted from each segmented neuron. (E) Spearman’s rho correlations between each neuron’s activity over the experimental condition are used to create the full functional connectivity matrix. As these matrices represent undirected networks, the graph is symmetrical along the origin (black boxes) with each half of the matrix redundant to the other. (F) A threshold is applied to the matrix to retain a proportion of the fully connected matrix (strongest 15% depicted). (G) Using a 15% proportional threshold, neurons with strong functional connectivity are connected by a line between somas (arbitrary colors). Analyses are performed on 2–4 separate network cultures per day.

To determine methamphetamine regulation of ventral midbrain neuronal networks culture, ventral midbrain was extracted from P0-P1 C57B6/J mice and transduced with the GCaMP6f transgene under a general neuronal promoter (Fig 2A). We monitored spontaneous calcium activity in a 10x field of view at 10 Hz (Fig 2B). Neuronal cell bodies were manually segmented and spontaneous calcium activity of GCaMP6f expressing neurons was extracted in order to calculate functional connectivity between neurons (Fig 2C and 2D). Symmetrical undirected adjacency matrices that represent co-active neurons were generated using the Spearman’s rank correlation coefficient (Spearman’s rho) of calcium activity signals before and during methamphetamine exposure (Fig 2E). Proportional thresholds were applied to isolate and explore the strongest 15% of functional connections and network topology at baseline and during methamphetamine exposure (Fig 2F). Connections surviving the threshold are then projected onto the recording to investigate the spatial location of the connected neurons (Fig 2G). Surprisingly, the strongest connections were not spatially localized but rather comprised by both proximal and distal neurons.

**Fig 3:**
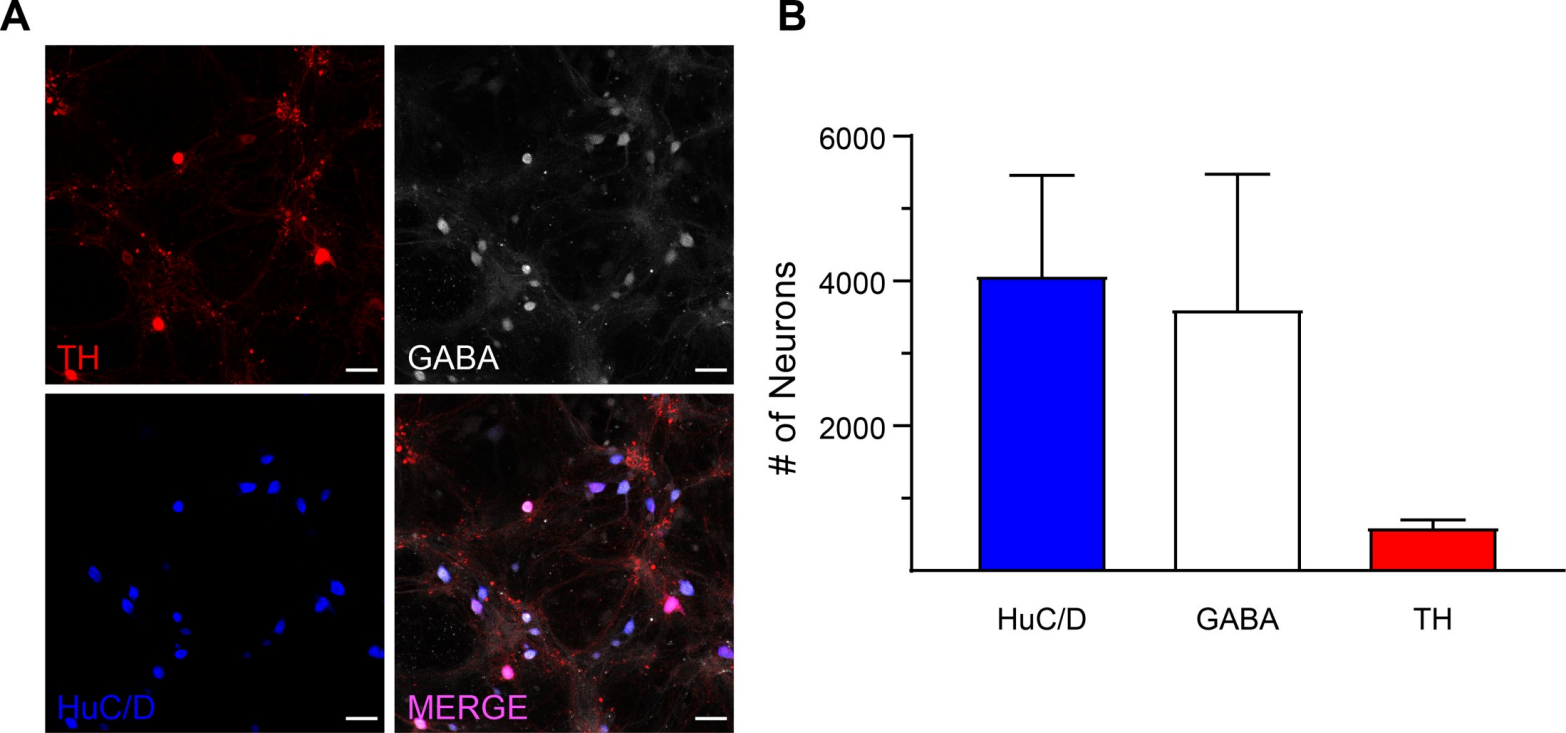
Characterization of neurons in our model system. (A) Neurons are identified immunohistochemically by HuC/D (a general neuronal marker–blue, mean = 4085 ± 1419 from 7 independent replicates), GABA (neurotransmitter product of GAD65 and GAD67 –white, mean = 3619 ± 1900, from 3 independent replicates), or tyrosine hydroxylase (rate-limiting enzyme of dopamine synthesis–red, 609 ± 134, from 6 independent replicates). Scale bar = 40 μm. (B) GABAergic neurons are the predominant neuronal type in ventral midbrain neuronal culture. Data are presented as mean ± SD.

**Fig 4:**
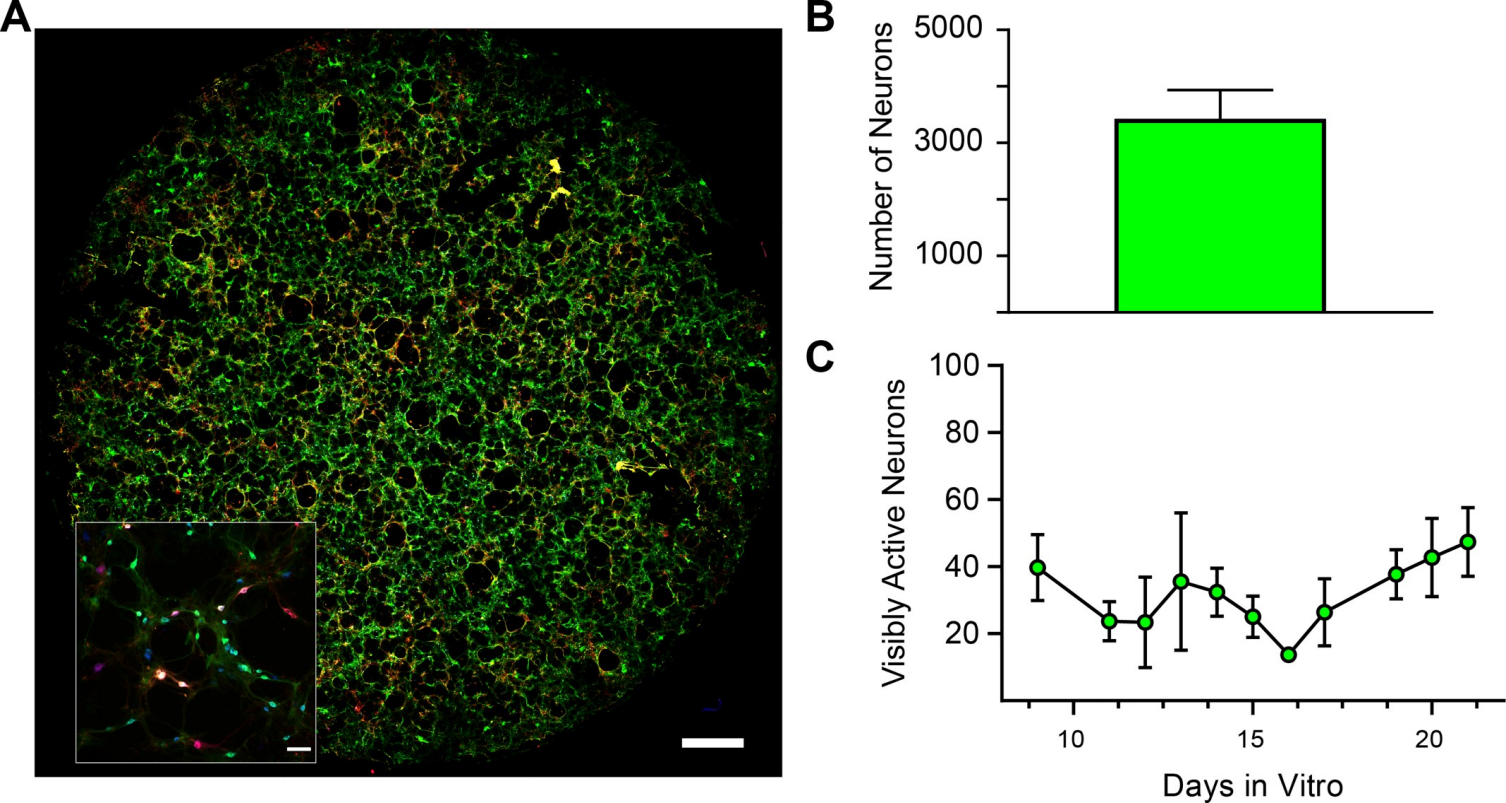
Length of neuronal culture does not affect number of neurons in our model system. (A) Representative image of ventral midbrain neurons. Scale bar = 1000 μm. Inset shows neurons from representative image stained for GCaMP6f (green), tyrosine hydroxylase (red), HuC/D (blue). Inset scale bar = 75 μm. (B) Coverslips are comprised of approximately 3400 neurons. Data are presented as mean ± SD. (C) No significant change in visibly active neurons across all days (simple linear regression, p = 0.0916, r^2^ = 0.08383; mean ± SD, from 3 independent replicates per day).

The ventral midbrain is a heterogeneous region comprised of neurons that vary in their neurotransmitter content and protein expression [7]. Therefore, we first quantified the ventral midbrain neuronal network cultures for the presence of tyrosine hydroxylase (TH, the rate-limiting enzyme of dopamine synthesis), gamma-aminobutyric acid (GABA), and HuC/D (an RNA-binding protein, as a pan-neuronal marker) (Fig 3A). TH labelling revealed that dopaminergic neurons, a direct target of methamphetamine, comprise approximately 12% of the total neural network (relative to HuC/D), with the remaining neurons being predominantly GABAergic (Fig 3B). These GABAergic neurons are one of the main targets of the released dopamine from dopaminergic neurons during methamphetamine exposure. Ventral midbrain neurons have been extensively characterized at the single neuron level [27–30], but there is no established standard for studying ventral midbrain neuronal networks under basal condition and during methamphetamine exposure. Therefore, to establish a standard methodology for network analysis *in vitro*, the experiments and analysis were performed across development (nine to twenty-one days of neuronal network culture). We found the majority of neurons (~84%, mean HuC/D = 4085 ± 1419, mean GCaMP6f = 3426 ± 507) express GCaMP6f were similar amongst experimental groups and across development (denoted by Days *In Vitro* (DIV) Fig 4A and 4B). As we were interested in the temporal relationship between DIV and network dynamics, we first controlled for the temporal relationship between DIV and the number of recorded neurons per experimental group. Therefore, we assessed this relationship using simple linear regression and found no significant relationship (n = 3 independent replicates per DIV, p = 0.0916, r^2^ = 0.08383), even when DIV is treated as categorical and assessed through one-way ANOVA (n = 3 independent replicates per DIV, p = 0.1378, F (11, 23) = 1.695) (Fig 4C). These data establish a reliable model system that can be employed to examine the functional connectivity and topology of ventral midbrain neuronal networks culture and stem cell-derived human neuronal networks. By establishing this model, it will be possible to relate how the dysfunction of a single neuron can establish pathological spreading of dysfunction that is believed to underly disorders of the ventral midbrain such as Parkinson’s disease [31]. Furthermore, determining how the network responds to change, either through drug exposure or disease state, our model provides the first step towards understanding how changes in neuron-to-neuron communication underlie more complex responses. It should be noted that the cultured network model is expressly removed from both inputs and outputs that are maintained from a regionally intact system, such as an acute brain slice, and therefore could be used to investigate intrinsic and self-organizing principles that would be reconciled in model system receiving multiple overlapping inputs. Future experiments will be necessary to elucidate differences between ventral midbrain network cultures and midbrain slice preparations.

### Methamphetamine suppression of network activity is D_2_ receptor dependent

**Fig 5:**
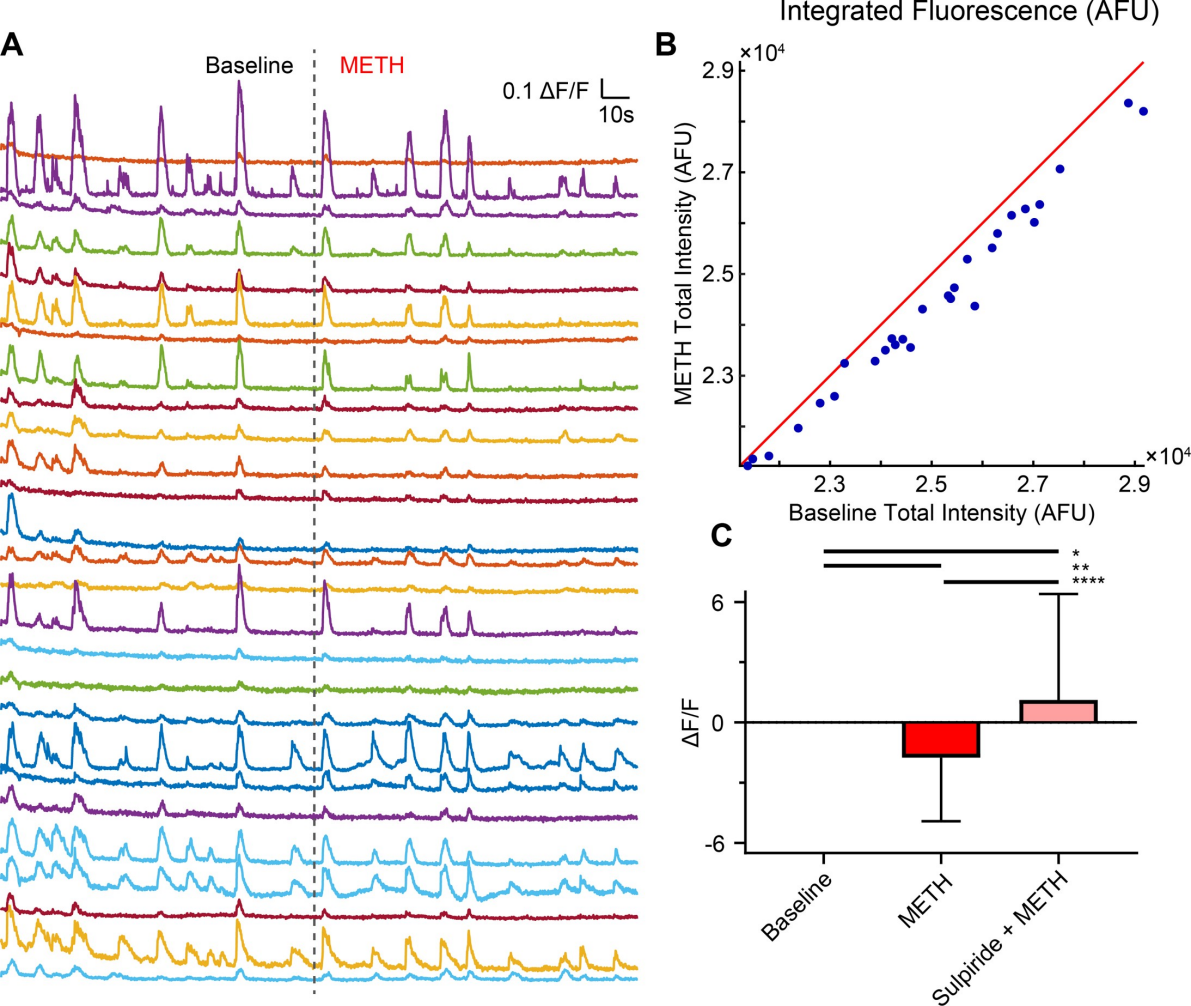
Methamphetamine exposure decreases midbrain neuronal activity via a D_2_ receptor mechanism. (A) Calcium activity of neurons is monitored at baseline and during methamphetamine addition (dotted line). (B) Scatter plot of the total intensity per neuron (blue dots) showing activity skewing below unity (red line) to the baseline condition, indicating activity is suppressed. (C) Methamphetamine-induced increases in dopamine may suppress the activity of GABAergic neurons through D_2_ receptor activation. Methamphetamine-induced suppression of integrated fluorescence analysis is blocked by D_2_ receptor antagonism (sulpiride, 5 μM) leading to increased network activity (One-way ANOVA *p< 0.05, **p<0.01, ****p<0.0001, F (2, 307) = 14.03; n = 3–4 independent replicates per experimental group; mean ± SD).

Increases in action potential frequency have been shown to correlate with increased amplitude and duration of GCaMP6f fluorescence signal [32]. As such, integration of fluorescence signals is commonly used to estimate neuronal activity [33–35]. To determine whether methamphetamine differentially affects the activity of individual neurons as well as the overall network output, the spontaneous calcium signals at baseline were integrated and plotted against the spontaneous calcium signals during methamphetamine treatment. Comparing the neuronal activity of each neuron before and during methamphetamine treatment, we found during methamphetamine exposure, the activity of all neurons skews below unity, suggesting methamphetamine decreases the overall activity of the network (Fig 5B and 5C) (One-way ANOVA *p< 0.05, **p<0.01, ****p<0.0001, F (2, 307) = 14.03; n = 71–125 neurons from 3–4 coverslips per group; mean ± SD). These data are consistent with the hypothesis that methamphetamine not only decreases activity of individual GABA neurons but also manifests across the network. We reasoned that methamphetamine induces network suppression, theoretically, through modification of synaptic signaling.

Methamphetamine has been shown to increase extracellular dopamine levels via two different mechanisms: increased firing activity of dopamine neurons and via the dopamine transporter-mediated dopamine efflux–the action potential-independent mechanism of dopamine release [22,29,36]. The released dopamine binds to dopamine receptors on the neighboring neurons to modulate their activity [4,5]. D_2_ receptors are negatively coupled to adenylyl cyclase, and agonist activation of D_2_ receptors decreases neuronal activity [37]. The ventral midbrain GABAergic neurons [16], which are the principal neuronal type in our model system, express D_2_ receptors [7]. Therefore, methamphetamine-stimulation of dopamine release is predicted to activate D_2_ receptors on these GABAergic neurons, leading to network suppression. Thus, we hypothesized that methamphetamine suppression of ventral midbrain neuronal network activity is mediated through a D_2_ receptor-dependent mechanism. We tested this hypothesis by examining ventral midbrain neuronal network activity in the presence or absence of D_2_ receptor blockade (sulpiride, 5 μM), before and during methamphetamine treatment. Our analysis revealed that blockade of D_2_ receptor inhibited the methamphetamine suppression of neuronal network activity (Fig 5C). Importantly, we found that sulpiride blockade of D_2_ receptors not only inhibited methamphetamine suppression of network activity but also increased network activity above baseline (n = 71 to 134 neurons, from 6 independent replicates, one-way ANOVA with Tukey’s post-hoc multiple comparisons, p < 0.05, F (2, 307) = 14.03. All comparisons performed at DIV 11.). These data are consistent with the interpretation that methamphetamine-induced suppression of network activity is D_2_ receptor dependent, and ventral midbrain cultured neuronal networks are under inhibitory regulation of D_2_ receptor at baseline. Dopaminergic terminals contact adjacent and distal GABAergic cells [38,39]. These contacts provide the structural basis for dopamine modulation of individual synapses locally and the interconnection distal networks at baseline under physiological conditions. It should be noted that at the cellular level, the intrinsic excitability and synaptic efficacy of a single neuron is always under concomitant modulation of multiple neuromodulators. Therefore, reconfiguration of neural network by neuromodulators is an intricately balanced process that involves multiple synergistic or antagonistic pathways. The current study does not address these complex interactions. Bridging multiple levels of analyses including the activity of single neuron, local and distant networks can address these possibilities.

### Methamphetamine does not modify changes in ventral midbrain neuronal network functional connectivity but alters topological structure

**Fig 6:**
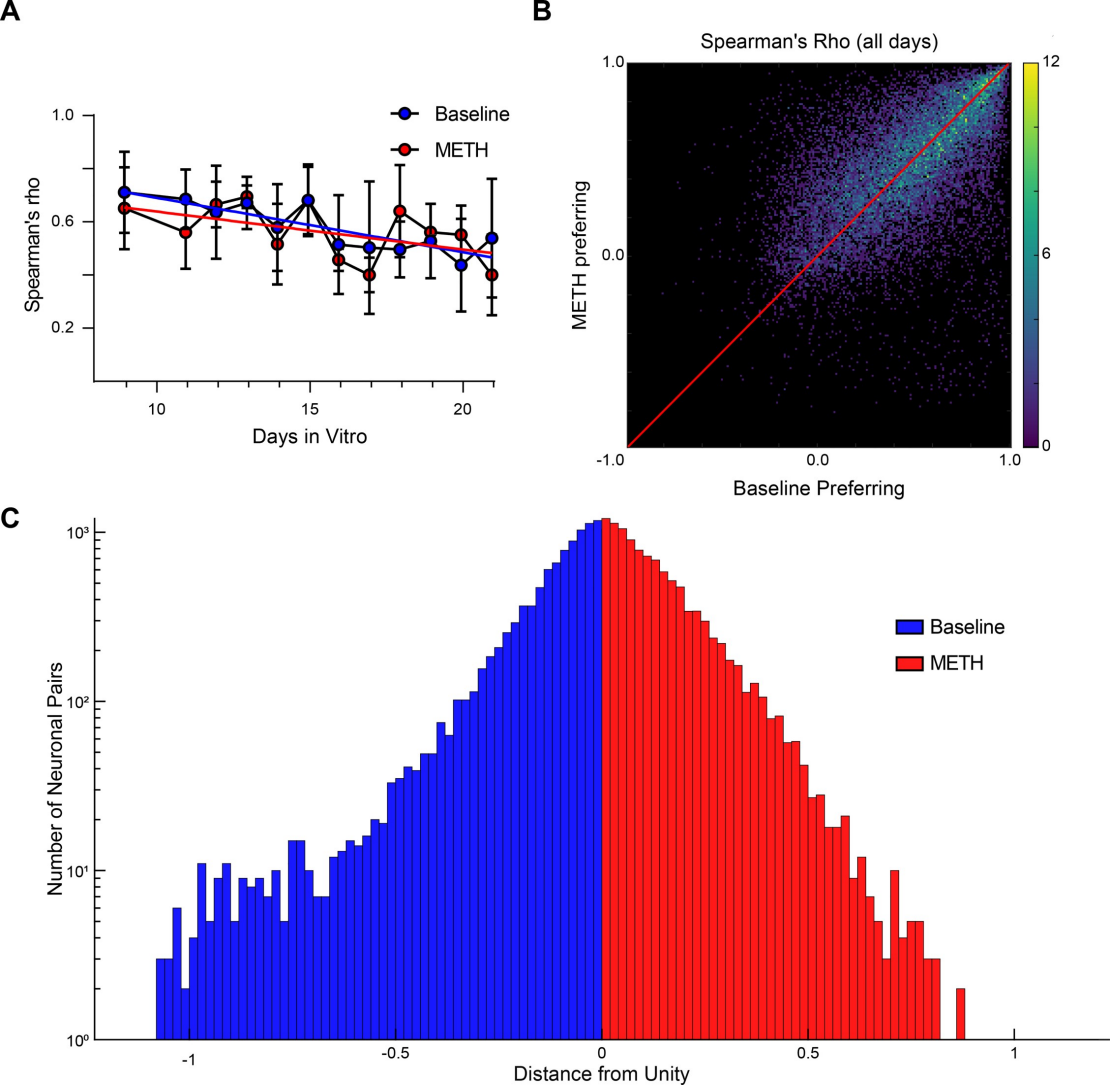
Time-dependent decay of functional connectivity is not affected by methamphetamine exposure. (A) Despite being highly active, functional connectivity of neuronal networks decayed significantly with network age and persisted in the presence of methamphetamine (simple linear regression; baseline p < 0.01, METH p < 0.05; n = 3 independent experiments per DIV; mean ± SD). (B) Three-dimensional histogram of functional connectivity in baseline versus methamphetamine between all neurons across all days reveals an asymmetric distribution around unity (red line) skewing towards baseline. (n = 20,216 pairwise connections, from 35 independent experiments). (C) Deviations from unity were compared between baseline-favoring (below unity) pairwise neuronal correlations and methamphetamine-favoring (above unity) pairwise neuronal correlations.

So far, our data suggest that methamphetamine suppresses ventral midbrain cultured neuronal network activity. However, it remains unknown whether methamphetamine modulates functional connectivity. Furthermore, the relationship between development and functional connectivity in ventral midbrain neuronal networks has not been studied. Previous reports investigating hippocampal and cortical neural networks have shown that functional connectivity increases across development [12,14]. Therefore, we investigated whether the functional connectivity of ventral midbrain neuronal networks is also altered by development and if methamphetamine modulates this potential development-dependent regulation of ventral midbrain neuronal networks. We found, in the absence of drug treatment (baseline), that there is a significant decrease in the functional connectivity across network development with an average of 0.71 at DIV 9 (DIV: Days *In Vitro*) to 0.54 on DIV 21 with a decay of 0.02 per day (simple linear regression, p < 0.01, r^2^ = 0.2098, n = 3 independent experiments per DIV) (Fig 6A, black). These data are specifically important because the total number of neurons, the number of active GCaMP6f neurons, and thus network size, did not change across days (Fig 4C). Therefore, the age-dependent decreases in the functional connectivity suggest a developmentally related (fine tuning) maturation or elimination of the network. However, since networks were of similar size, it is not clear whether this observed phenomenon is dependent on the total number of neurons observed within the network.

During methamphetamine exposure, similar decreases in functional connectivity of the network across development were observed with an average of 0.65 at DIV 9 and 0.40 at DIV 21 with a decay 0.014 per day (simple linear regression, p < 0.05, r^2^ = 0.1394, n = 3 independent experiments per DIV) (Fig 6A, red). This suggests that the observed changes in functional connectivity are robust against acute methamphetamine exposure. Though methamphetamine exposure has previously been shown to exert pathological effects [40,41], these were not observed to directly manifest at the level of average connectivity. These data suggest methamphetamine did not alter average functional connectivity across developmental days studied here; therefore, we hypothesized that methamphetamine had unevenly altered a subset of connections–for example, strong connections get stronger while weak connections get weaker–that may be obfuscated by average measurements. To address this hypothesis, we generated a connection value density plot during baseline and during methamphetamine treatment (Fig 6B). Connectivity was observed to be predominantly positive in baseline and during methamphetamine exposure. However, the distribution around the unity line appeared to be uneven, with highly connected neuronal pairs from baseline dropping out and becoming negatively connected in response to methamphetamine. To evaluate this, a histogram was generated to evaluate the distance from the unity line for each neuronal pair (Fig 6C). The magnitude of the measurement quantifies the strength of change between condition with positive values, indicating large changes in favor of the methamphetamine condition and negative values favoring the baseline condition. The distribution was found to show non-normal distribution (Kolmogorov-Smirnov, p < 0.05, n = 20,216 pairwise connections, from 35 independent experiments) with a significant, but modest, positive mean value (Wilcoxon signed rank, p < 0.01, n = 20,216 pairwise connections, from 35 independent experiments). From the histogram, negative skewing was readily apparent (skewness = -0.5417, n = 20,216 pairwise connections, from 35 independent experiments) and further kurtosis testing, a tail-distribution metric, revealed stronger than normal contribution (kurtosis = 6.3653, normal distribution kurtosis = 0, n = 20,216 pairwise connections, from 35 independent experiments). This finding reveals that a significant subset of connections dropout during methamphetamine exposure. As the network is predominantly GABAergic (~88% Fig 3B), this suggests that non-GABAergic neurons are most likely to be those that dropout. This implies that changes in connectivity are particularly nuanced. Future studies will be necessary to identify the specific neuronal phenotypes implicated in these changing connections, whether they be dopaminergic-dopaminergic, GABAergic-dopaminergic, or GABAergic-GABAergic connections.

**Fig 7:**
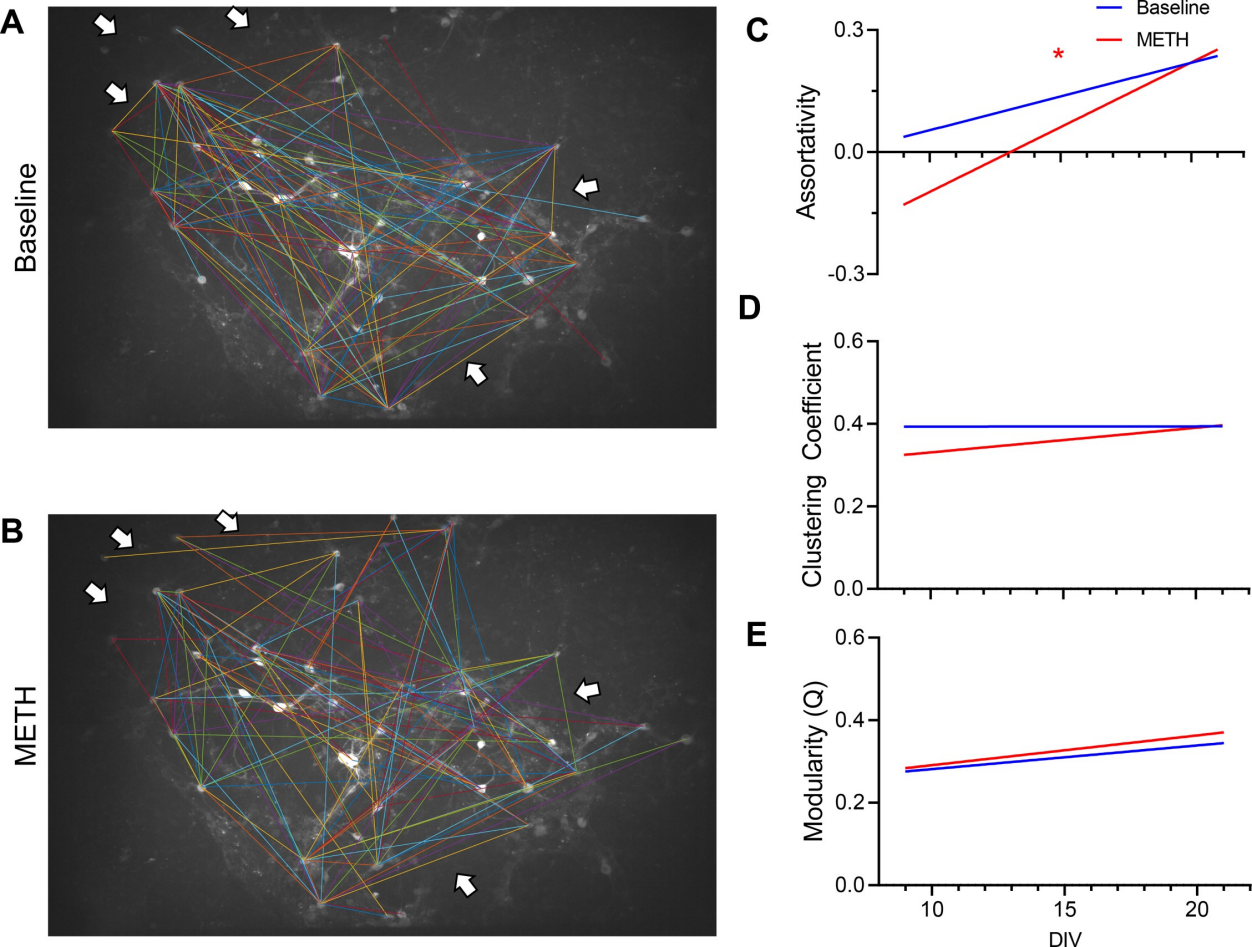
Methamphetamine and age of cultured network modulate functional network topology. (A,B) To minimize differences in network size, 15% proportional thresholds were applied during baseline (A) and in the presence of methamphetamine (B) to emphasize connection gains/losses (white arrows). (C) Network assortativity increases significantly across days (Two-factor ANOVA p < 0.01, F (11, 46) = 3.669, 38.51% of total variation, n = 3 independent experiments per DIV) but not between baseline and methamphetamine (p = 0.1078, F (1, 46) = 2.690, 2.566% of total variation, n = 3 independent experiments per DIV). No significant interaction was observed between DIV and condition (p = 0.1750, F (11, 46) = 1.472, 15.45% of total variation, n = 3 independent experiments per DIV). While increases across DIV at both baseline and in the presence of methamphetamine, only methamphetamine reached statistical significance (linear regression, p < 0.01, r^2^ = 0.2218 METH, p = 0.0988 r^2^ = 0.08042 Baseline, n = 3 independent experiments per DIV). Data presented as lines of best fit from simple linear regression. (D, E) To investigate interconnectivity and community restructuring, clustering coefficients and modularity index (Q) were calculated across DIV and conditions, but no significant trends or effects were observed. Data presented as lines of best fit from simple linear regression.

The absence of change(s) in the functional connectivity may or may not coincide with change(s) in the network structure. Therefore, we sought to identify and quantify the methamphetamine-modulation of ventral midbrain neuronal network topology. We applied a commonly used 15% proportional threshold [42–44] to isolate the strongest 15^th^ percentile of connections before and during methamphetamine exposure. This analytical approach revealed a qualitative rearrangement of a connectivity in the strongest connections (Fig 7A and 7B). These data are consistent with the hypothesis that topological features of a methamphetamine treated network diverge from their drug-naïve counterpart. In order to further investigate this hypothesis, we applied a weighted assortativity measure and found that assortativity significantly increased across development. While both baseline and methamphetamine conditions increased across days in culture (Two-factor ANOVA p < 0.01, F (11, 46) = 3.669, 38.51% of total variation, n = 3 independent experiments per DIV), only the methamphetamine condition reached significance criteria (simple linear regression, p < 0.01, r^2^ = 0.2218, n = 3 independent experiments per DIV) (Fig 7C). The observed increase in assortativity ranged from negative assortativity on DIV11 that converted to positive assortativity starting at DIV14, continually increasing up to DIV21. These data suggest that, as observed in previous studies, communication in younger cultured networks is more vulnerable to neuronal loss during methamphetamine exposure, while that in older cultured networks are more resilient. However, it is not directly clear why the network drastically reorganized in the early culture age and, specifically at which level this is directly modulated across culture age. In addition, network maturation may provide a compensatory refinement of connections that favors positive assortativity and reduces the impact of methamphetamine-induced toxicity [45–48]. Future experiments may reveal whether there are changes in molecular expression patterns, receptor interactions, or structural connectivity changes that may underlie this observation.

In addition, we analyzed other common metrics in network analysis such as network clustering or modularity. Clustering coefficient determines the interconnectedness, whereas, modularity index is an efficiency metric describing how well the clusters are classified into subnetworks. We found that neither development nor acute methamphetamine exposure affected network clustering or modularity (Fig 7D and 7E) (two-way ANOVA with Sidak’s multiple comparisons test, p > 0.05, F (11, 46) = 0.3424 clustering, F (11, 46) = 0.7777 modularity, n = 3 independent experiments per DIV). Though non-significant, there may be apparent trends, e.g. trend-wise increases in modularity across both conditions and initial decreases in clustering that were negligible in aged cultures, that were not uncoverable in our sample sizes or range of culture age. In addition, we also analyzed the D_2_ receptor dependencies for network clustering, modularity and assortativity metrics but did not observe any significant effects on these measures (S1 Fig, p > 0.05, F (2, 7) = 1.767clustering, F (2, 7) = 0.08639 modularity, F (2, 7) = 1.601 assortativity, n = 3–4 independent experiments per group. All comparisons performed at DIV 11). These data are consistent with the interpretation that methamphetamine exposure alters cultured network assortativity independent of D_2_ receptor activity. While these results were unexpected, they illustrate that the effects of methamphetamine scale from neurons to the network. Furthermore, the D_2_ receptor activity was not found to be responsible for changes in network structure and function. Rather, methamphetamine exposure leads to perturbations in multiple modalities, such as receptors, intrinsic neuronal properties, and neuroimmunological factors, that may scale to the network topology.

The major findings in terms of network reorganization in this study were a loss of neuron pairs in response to methamphetamine (Fig 6) and the demonstration that younger cultures are more vulnerable to neuronal loss during methamphetamine exposure, resulting in dramatic reorganization. These effects can be explained through “symmetry breaking” in a network. Broadly, in a symmetric network, each neuron is, at the least, reciprocally connected to each other. The extreme condition is where each neuron is connected to every other neuron. In this example, should one cluster of neurons become active, it quickly propagates and disperses. However, symmetric breaking, which can occur through pruning, enhancement of synapses, etc., provides the mechanism by which undirected activity propagation in the symmetric network can become directed and rhythmic [49,50]. Activity has a favored direction of flow which is reinforced every time a pathway is taken, further enhancing the asymmetry. Therefore, the vulnerability of younger networks to methamphetamine exposure potentially speaks to a lack of symmetry breaking in the network. Simply, not enough time has passed to favor certain connected pathways over others. Similarly, the dropping out of neurons during methamphetamine exposure suggests that the network converges on a minority of potential asymmetric pathways. Simply stated, methamphetamine reduces the repertoire of potential pathways that the activity can move, providing a network-level description on the neurobiological basis of addiction.

The work presented here represents the first foray, to our knowledge, in determining the mechanisms of ventral midbrain network modulation by methamphetamine. Ventral midbrain networks were found to exhibit development-specific changes in functional connectivity and topological changes during methamphetamine exposure. While this study attempted to isolate D_2_ receptor-dependent differences, further studies are warranted to determine interaction effects between multiple receptor types and subtypes. Systematic blockade of receptors may provide clues into the polypharmacy necessary to reverse the effects of methamphetamine exposure that are not readily observable using previously established methodologies.

Establishing this methodology provides a unique framework to explore ventral midbrain networks across multiple modalities. For example, using dopaminergic enrichment methods [51] permits the evaluation of dopaminergic contributions to network structure and function. These can be coincided with simultaneous imaging of stress indicators and network electrophysiology when grown on microelectrode arrays to provide both high temporal resolution of neuronal activity and correlative changes in neuronal stress.

This study examined the effects of acute methamphetamine exposure, and further studies can utilize repeated exposure paradigms across development, thereby not only tracking network evolution but also whether and how methamphetamine exposure modifies this. Previous studies investigating networks enriched in dopaminergic content [42,43,52] have focused on whole-brain interactions that do not uncover differences at the single-neuron resolution. Differences at the single-neuron resolution may compound upon repeated exposures and yield progressive transitions across the network that precede the onset and underly the establishment of untoward consequences of methamphetamine exposure. Ultimately, this study provides the fundamental basis for the continued exploration of ventral midbrain network modulation and diseases associated with dopaminergic dysfunction.

## Materials and methods

### Animals

Wild-type (WT) C557BL/6J male and female mice were obtained from The Jackson Laboratory (Bar Harbor, Maine). All animals were maintained in the University of Florida animal facilities. All experiments were approved by the Institutional Animal Care and Use Committee at University of Florida.

### Primary ventral midbrain culture

Full protocol for preparation is available at: https://www.protocols.io/private/C16AFE64BBD572063A78466BB79CCDDD

Briefly, dissociated post-natal ventral midbrain cells from C57BL6/J mice of either sex comprising neurons and glia were plated at 80,000 cells per coverslip. Ventral midbrain cultures were grown in 2 mL Neurobasal Plus supplemented with B-27 plus, 2 mM L-glutamine, 1 ng/mL GDNF, 0.4 mM kynurenic acid. Media was exchanged every 4 days by removing 1 mL old media and adding 1 mL fresh media (without GDNF/kynurenic acid supplement).

### Viral transduction

Cultures prepared from C57BL6/J mice were transduced with AAV5.Syn.GCaMP6f.WPRE.SV40 (MOI: 30,000) on DIV 0. AAV5.Syn.GCaMP6f.WPRE.SV40 was a gift from The Genetically Encoded Neuronal Indicator and Effector Project (GENIE) & Douglas Kim (Addgene viral prep # 26973-AAV5). Neurons were imaged after sufficient expression was observed between DIV 9 and DIV 21.

### Calcium imaging

Briefly, mouse midbrain networks were transferred to the recording chamber and imaged under constant gravity perfusion of artificial cerebral spinal fluid (ACSF, Table 1) at 37C. A Spectra X equipped with 7 excitation lines configured with a Mightex Polygon400 Digital Micromirror Device was used to excite both GCaMP6f (λ_ex_ = 470 nm) through a quad-pass filter (Chroma Technologies, Brattleboro, Vermont), Nikon CFI Plan Fluor 10XW objective (NA = 0.3) and detected with an Andor Zyla 4.2 sCMOS 12-bit camera at 10 frames per second. Videos were acquired with a 2-minute baseline of spontaneous network activity in ACSF and a 2-minute experimental condition in ACSF, ACSF with 10 μM methamphetamine, or 10 μM methamphetamine and 5 μM sulpiride.

**Table 1:** Composition of ACSF.

ACSF (pH 7.4, 310 mOsm)			
Chemical	Concentration	Vendor	Catalog Number
NaCl	126 mM	Sigma-Aldrich	S7653-1KG
KCl	2.5 mM	Sigma-Aldrich	P9541-500G
CaCl2	2 mM	Sigma-Aldrich	223506-500G
NaH2PO4	1.25 mM	Sigma-Aldrich	71505-250G
MgSO4	2 mM	Sigma-Aldrich	M7506-500G
Dextrose	10 mM	Sigma-Aldrich	D9434-500G

### Image processing

Videos were imported into ImageJ2 via the Bio-Formats Importer plugin and converted into 8-bit TIFF stacks. Image drift was corrected using the moco plugin when necessary [53]. Regions of interest (ROI) for neuronal cell bodies were segmented manually and signals were exported into CSV files. Only visibly spontaneous active neurons with non-overlapping, distinguishable somas were segmented.

### Functional connectivity and quantification of network topology

CSV files were imported into MATLAB (Mathworks Cambridge, MA). Calcium signals were normalized and expressed as F(t) = ΔF/F_0_ where F_0_ is defined as the baseline condition. Pairwise Spearman’s correlation coefficients were determined over the entire 2-minute time series of each condition between neurons (MATLAB function: corr, type: Spearman). Densiometric measures were applied using a range of thresholds ranging from correlation values of 0.05 to 1 with 0.05 step-size and averaged by day and condition. To quantify changes in network topology of the strongest connections, a 15% proportional threshold retaining the 15% strongest connections were applied to each adjacency matrix. Applying thresholds also improved the ability to compare across networks that varied in size. Network clustering assortativity, clustering coefficient, and modularity were calculated using the Brain Connectivity Toolbox [9]. All calculations retained weighted values, and undirected weighted versions of the respective metrics were used where appropriate [54–56]. Importantly, these metrics are performed on activity networks with single-cell resolution and are not assumed to reflect synaptic connectivity. Furthermore, sampling rates were appropriate to investigate functional but not effective connectivity in these networks and are outside the scope of the current investigation. Future studies should investigate the synaptic and effective connectivity differences across ventral midbrain networks.

### Immunocytochemistry and network characterization

Immunocytochemistry–Cultures were washed three times in phosphate buffered saline (PBS), fixed in 4% paraformaldehyde in PBS, and washed three more times in PBS. Cultures were then blocked and permeabilized in 0.5% Triton X-100 and 10% normal goat serum (NGS) for 30 minutes at room temperature, followed by three washes in PBS. Cultures were incubated overnight in primary antibody solution containing mouse anti-TH (1:500), mouse anti-HuC/D (1:500), and rabbit anti-GFP (1:500) or rabbit anti-GABA (1:1000). Negative controls utilized antibody dilution solution without primary antibodies. Cultures were then washed three times for 20 minutes at room temperature on an orbital shaker. Then cultures were incubated in secondary antibody solution containing Goat Anti-Rabbit Alexa Fluor 488 conjugated, Goat Anti-Mouse IgG1 Alexa Fluor 568 conjugated, and Goat Anti-Mouse IgG2b Alexa Fluor 647 or Goat Anti-Rabbit Alexa Fluor 405 conjugated antibodies on an orbital shaker in the dark for one hour at room temperature. All antibodies were diluted with 5% normal goat serum in 0.1% Triton X-100 in PBS. Cultures were then washed three times for 20 minutes at room temperature on an orbital shaker in the dark. Finally, cultures were mounted on slides using either Fluoromount-G or DAPI Fluoromount-G and allowed to harden. Reagent catalog numbers are presented in Table 2.

**Table 2:** Immunocytochemistry reagents utilized in this study.

Chemical	Concentration	Vendor	Catalog Number
Phosphate Buffered Saline	N/A	N/A	N/A
Triton X-100	Varies	Fisher	BP151-100
Normal Goat Serum	Varies	Lampire Biological Products	7332500
Rabbit Anti-GABA	1:1000	Immunostar	20094
Rabbit Anti-GFP	1:500	Invitrogen	A-11122
Mouse Anti-Tyrosine Hydroxylase	1:500	Sigma-Aldrich	T1299
Mouse Anti-HuC/D	1:500	Invitrogen	A-21271
DAPI Fluoromount-G	N/A	Southern Biotech	0100–20
Fluoromount-G	N/A	Southern Biotech	0100–01
Paraformaldehyde	4%	Electron Microscopy Sciences	15714-S
Goat Anti-Rabbit IgG (H+L) Alexa Fluor 405	1:500	Invitrogen	A-31556
Goat Anti-Mouse IgG1 Alexa Fluor 568	1:500	Invitrogen	A-21124
Goat Anti-Mouse IgG2b Alexa Flour 647	1:500	Invitrogen	A-21242
Goat Anti-Rabbit IgG (H+L) Alexa Fluor 488	1:500	Invitrogen	A-11034

Imaging and Quantification–Coverslips were imaged in their entirety utilizing a Keyence BZ-X700 microscope with the stitching module through a Nikon CFI Plan Apo λ 10X objective, metal halide lamp, and Keyence standard filters for each channel. Stitched images of each channel were exported as single-channel TIFF images. TIFFs were imported into ImageJ2. Neuronal cell bodies were identified by HuC/D and or DAPI signal. GCaMP6f expressing cells were identified by GFP signal. Dopaminergic neurons were identified by TH signal. GABAergic neurons were identified by GABA signal. All neurons were counted manually.

### Statistical analysis

All statistical analysis was performed in Graphpad Prism version 8.02 for Windows, GraphPad Software, La Jolla, California, USA. Linear regression, one-way ANOVA, and two-way ANOVA were used where appropriate and corrected for multiple comparisons using either Tukey’s or Sidak’s post-hoc test. Effects were considered significant at α = 0.05. Data are presented with mean and standard deviation unless otherwise stated.

## Supporting information

S1 Fig Specific network topology modification by methamphetamine is not D2 receptor dependent. (A) To assess whether D_2_ receptor availability alters network function, similar age networks were exposed to either continued baseline or methamphetamine in the presence of sulpiride. Neither methamphetamine exposure nor sulpiride co-administration produced a significant change (One-way ANOVA, p = 0.2677, F (2, 7) = 1.601; mean ± SD). (B,C) Network clustering and modularity are unaltered by either exposure to methamphetamine alone or with co-administration of sulpiride (One-way ANOVA, p = 0.2392, F (2, 7) = 1.767, clustering coefficient; p = 0.9182, F (2, 7) = 0.08639, modularity; mean ± SD). Data are presented as mean relative change ± SD.
(TIF)
