# Supplementary figures and images for: Methamphetamine regulation of activity and topology of ventral midbrain networks
Source: PLoS One. 2019 Sep 19;14(9):e0222957. doi: 10.1371/journal.pone.0222957 (PMC6752877; doi:10.1371/journal.pone.0222957)

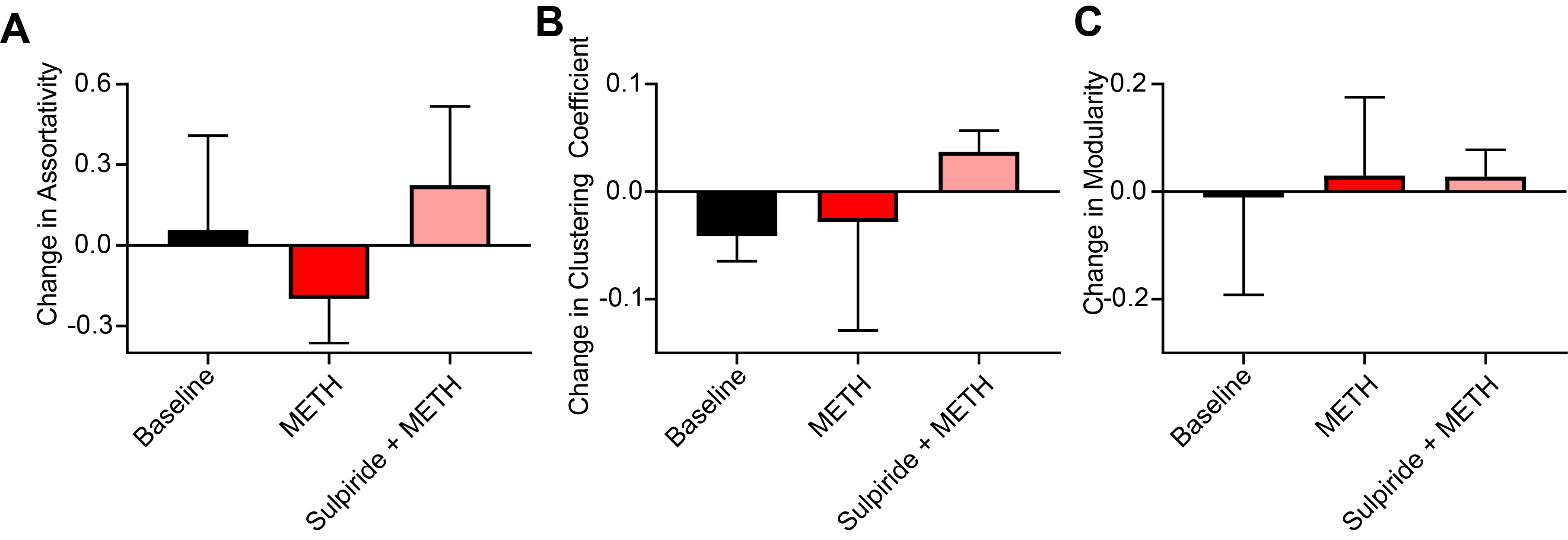

Supplement: S1 Fig — (A) To assess whether D2 receptor availability alters network function, similar age networks were exposed to either continued baseline or methamphetamine in the presence of sulpiride. Neither methamphetamine exposure nor sulpiride co-administration produced a significant change (One-way ANOVA, p = 0.2677, F (2, 7) = 1.601; mean ± SD). (B,C) Network clustering and modularity are unaltered by either exposure to methamphetamine alone or with co-administration of sulpiride (One-way ANOVA, p = 0.2392, F (2, 7) = 1.767, clustering coefficient; p = 0.9182, F (2, 7) = 0.08639, modularity; mean ± SD). Data are presented as mean relative change ± SD. (TIF) [file pone.0222957.s001.tif]
